# Energy, nutrients and food sources in snacks for adolescents and young adults

**DOI:** 10.1590/1984-0462/2022/40/2020148

**Published:** 2021-07-30

**Authors:** Tatiane dos Santos Lopes, Aline Veroneze de Mello, Luana Romão Nogueira, Ana Carolina Barco Leme, Regina Mara Fisberg

**Affiliations:** a Universidade de São Paulo, São Paulo, SP, Brazil.; b Centro de Excelência em Dificuldades Alimentares, Instituto de Pesquisa e Ensino em Saúde Infantil, São Paulo, SP, Brazil.; c Family Relations and Applied Nutrition, University of Guelph, Canada.

**Keywords:** Eating, Snacks, Adolescent, Young adult, Ingestão de alimentos, Lanches, Adolescente, Adulto jovem

## Abstract

**Objective:**

To evaluate associations between snacking and energy, nutrients and food source, and to identify the contribution of snacking across age, sex, weight status and lifestyle behaviors among adolescents and young adults.

**Methods:**

A sub-sample was calculated from the population-based cross-sectional study 2015-Health Survey of São Paulo (ISA-Capital). The survey “ISA-Nutrição” used a sample of non-institutionalized individuals aged >15 years. For this study, only adolescents (12-18 years old; n=418) and young adults (19-29 years old; n=218) were included. Snacks were identified, and their contribution to energy, nutrients, and food sources were calculated. Descriptive statistics and logistic regressions were used.

**Results:**

Participants experienced an average of 2.9±0.6 snacking occasions per day. Young adults consumed more energy from morning and night snacks, and adolescents, from afternoon snacks. The top three food sources on snacking contributed to 30.5% of energy: cookies (11.8%), sugar sweetened beverages (9.4%), sweets and other desserts (9.3%). Although results were non-significant, being a female (*Odds Ratio* [OR] 0.93; 95% confidence interval [95%CI] 0.36-1.49), meeting the physical activity recommendations (OR 0.75; 95%CI 0.25-1.25), and scoring higher for the healthy eating index (OR 0.88; 95%C 0.24-1.52) were all factors related to increased intake of snacks. Alternatively, overweight individuals (OR -0.54; 95%CI -1.00 to -0.08) consumed less snacks.

**Conclusions:**

Improving the quality of snacks should be considered in behavior-change strategies.

## RESUMO

**Objetivo:**

Avaliar associações entre lanches e consumo de energia, nutrientes e fontes alimentares e identificar seus contribuintes por idade, sexo, *status* de peso e comportamentos de estilo de vida entre adolescentes e jovens adultos.

**Métodos:**

Subamostra calculada a partir do estudo de base-populacional transversal Inquéritos de Saúde de São Paulo (ISA-Capital, 2015). O ISA-Nutrição utilizou amostra de indivíduos não-institucionalizados com idade >15 anos. Para este estudo, apenas adolescentes (12-18 anos; n=418) e jovens adultos (19-29 anos; n=218) foram incluídos. Os “lanches” foram definidos, bem como foram calculadas a contribuição de energia e nutrientes, e as fontes alimentares. Estatística descritiva e regressões logísticas foram utilizadas.

**Resultados:**

Em média foram realizados 2,9±0,6 lanches por dia pelos participantes. Jovens adultos consumiram mais energia nos lanches da manhã e noite, enquanto adolescentes, à tarde. As três principais fontes de alimentos nos lanches contribuíram em 30,5% para o total de energia: biscoitos (11,8%), bebidas açucaradas (9,4%), e doces e outras sobremesas (9,3%). Apesar de os resultados não serem significantes, ser do sexo feminino (*Odds Ratio* [OR] 0,93, intervalo de confiança de 95% [IC95%] 0,36-1,49), atingir as recomendações de atividade física (OR 0,75, IC95% 0,25-1,25), e ter alta pontuação no índice do total da qualidade dieta (OR 0,88, IC95% 0,24-1,52) resultou em aumento na ingestão de lanches. Alternativamente, ter sobrepeso (OR -0.54, IC95% -1.00 a -0.08) está relacionado a menores chances de consumir lanches.

**Conclusões:**

Estratégias para melhorar a qualidade dos lanches deveriam ser consideradas nas intervenções de mudança de comportamentos.

## INTRODUCTION

While non-modifiable (i.e., genetics and other biological) mechanisms are partly the blame, modifiable risk factors (e.g., eating behaviors) are considered the primarily root in the genesis of obesity.^1^ ^,^ ^2^ Obesity is a significant public health concern because of its increase prevalence and adverse effects on psychosocial and physical health.^3^ ^,^ ^4^ Adolescents and adults had a higher overweight prevalence and older adults were higher for being obese.^5^

 Evidence shows that Brazilian adolescents and young adults are failing to meet current dietary recommendations of healthy eating,^6^ being more pronounced among adolescents from low-income families, and with ethnic/racially diverse backgrounds.^7^ Over the past decades, practitioners and policy makers have been worried about the increase frequency of snacking, i.e., in-between meals, their daily energy intake, and food and beverages sources consumed as snacks. Adolescents consumed roughly one-third of total daily energy intake from snacks.^8^ In adults, snacking occasions and total energy intakes has been increasing, while substituting lean meats and dairy food sources has been observed.^9^ Therefore, considerable contribution of snacking to adolescents and adults’ total energy intake are needed to comprehend how snacks contribute to weight status and other lifestyle behaviors.^9^ ^,^ ^10^

 Snacking has been defined and measured in variety of ways. Most common definition is based on the intake of energy-dense food sources. The inconsistent definitions are crucial to recognize when interpreting the results on snack studies. A review has defined snacks into three main factors, to allow differentiation from the main meals:

1. consumers’ perceptions, considering main meals vs. other eating occasions as snacks;
2. time of consumption based on the time slots for breakfast, lunch, and, dinner, and the other times are considered snacks; and
3. energy intake, snacks should be <15% of total energy intake. Moreover, distinction must be made in terms of dietary composition.^11^ Differences between industrialized and developing countries in terms of snacking food sources have been shown, an increased intake on savory snacks and sugar-sweetened beverages (SSB). Alternatively, snacks can provide an opportunity to supplement the diet where foods in meals are not adequately consumed. Eating a fruit as a mid-morning snack is a good way to meet the recommended intake of fruits, along with those eaten at meals.^11^

There is a gap on studies that explore the percussion of snacking on food consumption, weight-status, and other lifestyle behaviors, especially among racial/ethnically diverse and low-income individuals. Additionally, most studies conducted have been concentrated on school-age children from high-income countries and in identifying intakes on particularly nutrients (e.g., added sugars and saturated fats), or on the intake of specific food groups (e.g., SSB and savory snacks), so, little attention has been given on understanding the contribution of snacks on diet for other age groups, at a population level, in low- to middle-income countries. Hence, this study aimed to evaluate associations of snacking with Brazilian adolescents and adults’ energy and nutrient intakes, as well as food sources. A second aim was to identify differences in the contribution of snacks to the population dietary intake across age groups, sex, weight status, and lifestyle behaviors.

## METHOD

Data from the 2015 Health Survey of São Paulo (acronym in Portuguese “ISA-Capital”) were used. The ISA-Capital is a population-based, cross-sectional survey that uses a multistage stratified area probability sample of non-institutionalized individuals, that provides nationally representative estimates of the population of the city of São Paulo, Brazil. Briefly, the survey examined approximately 3,300 individuals in 2003 (n=3,357), 2008 (n=3,271), and 2015 (n=4,059). Data were collected by trained interviewers using a questionnaire, divided into sixteen blocks; of these, only two were used: health-related behaviors, including eating, physical activity (PA) and sedentary behaviors; and socio-demographic characteristics.

 In order to collect information about diet and nutrition, a sub-sample from the ISA-2015 was calculated. This sub-sample was named ISA-Nutrition (Health Survey of São Paulo with focus on Nutrition), in which 554 adolescents, 254 young adults, 390 adults, and 545 older adults randomly selected participated for a dietary data collection and further phases of the ISA-Nutrition. Additional details about this sub-sample study were previously published.^12^ The Institutional Review Boards from the School of Public Health, University of São Paulo and the Department of Health from the city Hall of São Paulo approved the ISA-Capital study, as well as the present study. Participants provided written informed consent and assent forms prior to their participation in the study.

The World Health Organization (WHO) defines adolescents as individuals aged between 10 and 19 years of age,^13^ and young adults have been defined in some studies^14^ as people aged 18 to 35 years. Therefore, data from adolescents and young adults aged 12 to 35 years old participating in the ISA-Nutrition were used, resulting in a final analytic sample of 636 (418 adolescents and 218 young adults) after exclusion of subjects not meeting this study criteria. They were excluded if their intake of foods and beverages were below or above their usual intake reported on 24h dietary recall (24hR), with the following questions asked: (a) “Were foods and beverages consumed yesterday the same as what you usually eat?” yes or no, why; and (b) Do you consider that the amount eaten yesterday was: (a) less (b) same (c) more than usually eaten? Individuals older than 30 years, pregnant women and lactating women, and individuals who did not report any food intake in the 24hR were also excluded. Socio-demographic information and self-reported physical activity levels were determined from the ISA-Capital survey. Physical activity was classified into two levels: meeting and not meeting the WHO guidelines for PA^15^ to the individuals’ intensity level in a typical week. The ISA-Capital employs protocols and procedures that ensure confidentiality and protect individual participant identification.

 Dietary data was collected from two 24hR in non-consecutive days, representing different week and weekend days and seasons of the year. The first 24hR was collected in home visits and the second with telephone interviews using the Automated Multiple Pass Method.^16^ The Nutrition Data System for Research (NDS-R, version 2014) was used to calculate energy and nutrients collected from the dietary recalls. As the NDS-R uses the United States Department of Agriculture food composition database, the energy and nutrient values provided in our study were compared to other Brazilian food composition databases.^17^

 Foods and beverages that most contributed to snack total energy-intake were identified and grouped based on nutritional values, eating frequency and other diet habits from individuals living in São Paulo. Foods and beverages consumed by less than 2% of the study sample were not grouped because of the high variability in terms of nutrient values. The energy contributing to each group was determined based on the method proposed by Block et al.^18^

 “Snack” was defined as the type of meal self-reported by participants. All participants reported snacking in ISA-Capital. Snacks were consistent with definitions previously published in the literature.^11^ Thus, this moment is considered an eating occasion other than breakfast, lunch, and dinner, which are known to take place commonly between 6-10am, 12-3pm, and 7-9pm, respectively, and it is comprised of all type of food and beverages, except water. Statistical modeling techniques incorporated in the Multiple Source Method (MSM) software were done to provide usual dietary intake adjusted for intra-personal variance of energy and nutrients intake, taking into account the information provided on the two 24hR. Sodium and added sugar were calculated as terciles of intake, classified as low, medium, and high.^19^ The overall diet quality of the sample was calculated based on the Brazilian Healthy Eating Index-Revised (BHEI-R),^20^ and categorized in terciles of intake.

 Height and weight were measured using procedures outlined in the ISA-Capital protocol.^12^ The body mass index (BMI) was calculated for adolescents according to BMI Z scores for age and sex: ≤+1 Z score were non-overweight and >+1 Z score were classified as overweight or obese. Young adults (≥19 years old) were classified according to the WHO’s standards, with BMI <24.99kg/m^2^ as non-overweight and BMI >25kg/m^2^ as overweight. Overweight and obese individuals were combined, like in previous studies.^21^

The International Physical Activity Questionnaire (IPAQ) long-version^22^ adapted for the Brazilian reality^23^ was assessed for PA duration, frequency, and intensity during leisure, work/school time, transportation, and household tasks. the information was dichotomized as meeting or not meeting WHO’s PA guidelines: adolescents (12-18 years old) should be at least 300 minutes/week and adults (≥19 years) at 150 minutes/week.^15^

 The time spent watching TV and using computers (or other screen devices, such as mobile phones, and tablets) was assessed in two questions. For each type of screen-time recreation, the participants were to report daily average hours during the weekdays and the weekends. The cut-off point for not being sedentary was less than two hours/day, according to the literature.^24^

Socio-demographic variables included sex, age group (adolescents vs. young adults), ethnicity (Caucasian and Non-Caucasian), educational level of the head of the family (middle school, high school, and some college/university degree), income status (≤1 and <1 minimum wage), smoking, and alcohol intake.

 Descriptive statistics were conducted using mean (standard error) for continuous variables, and frequency (% and 95%CI) for categorical variables. The Wald test was used to compare energy intake to socio-demographic and lifestyle behaviors, as well as the meal energy intake of participants. Multiple logistic regression models adjusted for the socio-demographic data and presence of chronic non-communicable diseases were performed to examine associations between socio-demographic features, weight-status, and lifestyle behaviors, including snacks intake. Snacks intake were categorized as ≤1 snack/day or >1 snack/day. All analyses were conducted in the software STATA 14.0 (Stata Corp, Texas, USA), with sample weights considering the complex survey design and with significance levels set at p<0.05.

## RESULTS

Adolescents and young adults in the study sample were between 12 and 29 years of age. The study sample (n=636) was composed by 52.6% of boys, most of them being non-Caucasian (59.1%), and 53.7% receiving less than 1 minimum wage/month, i.e., considered vulnerable in terms of socio-economic status (SES). As for the head of the family education background, most of them had only basic education degree (43.7%), followed by high-school (35.7%) and higher education (20.6%). Almost 70% of the participants were not overweight/obese, 71.1% of them did not meet the recommendations for PA and 87.9% of them spent more than 2 hours/day on screens. Other lifestyle behaviors, such as smoking (or former smoking) were seen only in 11.9% of the sample, while alcohol intake was present in 24.6% of the population studied. Additional details are shown in Table 1.

**Table 1:** Socio-demographic and lifestyle characteristics among adolescents (n=418) and young adults (n=218) from the 2015 ISA-Capital study.

Characteristics	n	%	95%CI
Sex			
Males	330	52.6	48.0-57.2
Females	306	47.4	42.9-52.0
Age group (years)			
12-18	418	51.7	47.2-56.1
19-29	218	48.4	44.0-52.8
Ethnicity			
Caucasian	254	40.9	36.0-45.9
Non-Caucasian	375	59.1	54.1-64.0
Income per capitaa			
≤1 MW	339	53.7	47.1-60.2
>1 MW	161	31.2	26.0-37.0
NR	97	15.1	9.8-22.5
Educational level of the head of the family			
Middle school	282	43.7	38.1-49.5
High school	201	35.7	31.0-40.7
Higher education	112	20.6	16.1-26.0
Weight status			
Normal weight	432	69.9	65.2-74.2
Overweight or obese	184	30.1	25.8-34.8
Physical activity level			
Does not meet recommendation	465	71.2	66.6-75.4
Meets recommendation	166	28.8	24.6-33.4
Time spent on screens			
≤2 hours/day	78	12.0	9.3-15.5
>2 hours/day	558	88.1	84.5-90.7
Smoking habits			
Never smoke	572	88.1	84.9-90.8
Former smoker or smoker	62	11.9	9.2-15.2
Alcohol intake			
Does not drink	517	75.4	70.3-79.8
Drink	117	24.6	20.2-29.7

^a^Minimum wage in the year of 2015 in Brazil was R$788,00 (Brazilian currency). The conversion rate at the time was 1 US Dollar=3,00 Brazilian Reais. 95%CI: 95% confidence interval; NR: No reply; MW: monthly wage.

**Figure 1:**
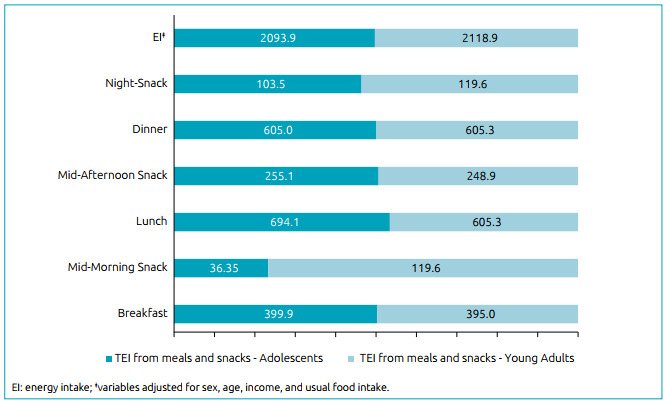
Average energy intake^‡^ from meals and snacks among adolescents (n=418) and young adults (n=218) from the 2015 ISA-Capital study.

 Young adults have a slightly higher energy intake (EI) (2118.9kcal/day) than adolescents (2093.9kcal/day), but there were no significant differences. Young adults have a significant higher EI in mid-morning (119.6kcal/day) and night (119.6kcal/day) snacks, with 36.4 vs. 103.5kcal/day for adolescents. However, adolescents have a significant higher EI in mid-afternoon snacks (255.1kcal/day) compared to young adults (248.9kcal/day) (Figure 1).

Participants had on average 2.9±0.2 snack times per day; each occasion contributed to a mean of 38.3±0.3kcal in morning snacks, 252.0±1.2kcal in afternoon snacks, and 111.5±2.1kcal in night snacks. Males have a significant higher energy intake in afternoon snacks (247.5±1.2 vs. 256.1±1.9 females) and night snacks (130.1±3.0 vs. 90.9±2.2kcal/day females), but females showed a higher energy intake in morning snacks (39.3±0.4 vs. 37.3±0.4kcal/day males). Young adults showed higher intake in morning snacks (40.2±0.5kcal/day) and night snacks (103.5±0.9kcal/day) compared to adolescents showing respectively, 36.4±0.2 and 103.5±2.2kcal/day. Adolescents had a significant higher intake in afternoon snacks (255.1±1.4 vs. 248.9±1.9kcal/day). Individuals who spent more than two hours on screens showed an increased energy intake in afternoon snacks (253.2±1.1 vs. 243.4±4.9kcal). There were no significant differences between weight status and physical activity as for energy intake in snack occasions. Data is available upon request for details on snack occasions in relation to socio-demographic features and lifestyle behaviors.

Table 2 shows snacks presenting nutrients of public concern, with mean differences between subjects. Young adults vs. adolescents had a significant higher intake of carbohydrates (1057.3 vs. 1008.1kcal), proteins (362.9 vs. 319.2kcal), total fats (712.0 vs. 678.2kcal), fiber (15.1 vs. 14.5grams), calcium (830.2 vs. 607.8mg) and sodium (3602.8 vs. 3238.0mg). Added sugars and iron intake was higher among adolescents than among young adults, respectively, 284.7 vs. 271.9kcal and 37.7 vs. 20.9mg.

**Table 2:** Differences in mean (95%CI) of macro and micronutrients in snacks by adolescents (n=418) and young adults (n=218) living in the city of São Paulo. 2015 ISA-Capital.

	Total Sample		Adolescents		Young adults	
**Carbohydrates,** mean (95%CI)						
g	257.7	(253.7-261.7)	252.0	(247.2-256.8)	**264.3**	**(257.9-270.8)**
kcal	1030.8	(1014.7-1046.9)	1008.0	(988.8-1027.2)	**1057.3**	**(1031.6-1083.1)**
**Proteins,** mean (95%CI)						
g	84.8	(82.9-86.8)	79.8	(77.5-82.0)	**90.7**	**(87.8-93.7)**
kcal	339.4	(331.5-347.3)	319.2	(310.1-328.2)	**362.9**	**(351.3-374.6)**
**Total fat,** mean (95%CI)						
g	77.1	(75.6-78.5)	75.4	(73.6-77.1)	**79.1**	**(76.8-81.4)**
kcal	693.8	(680.8-706.9)	678.2	(662.4-693.9)	**712.0**	**(691.6-732.5)**
**Added sugars,** mean (95%CI)						
g	69.7	(68.9-70.5)	**71.2**	**(70.4-72.0)**	68.0	(66.8-69.2)
kcal	278.8	(275.8-281.9)	**284.7**	**(281.4-288.1)**	272.0	(267.1-276.8)
**Fiber (g)**, mean (95%CI)	14.8	(14.5 -15.0)	14.5	(14.2-14.8)	**15.1**	**(14.8-15.5)**
**Calcium (mg)**, mean (95%CI)	710.1	(689.4-731.6)	607.8	(588.4-627.3)	**830.2**	**(802.9-857.5)**
**Iron (mg)**, mean (95%CI)	30.0	(27.7-32.3)	**37.7**	**(35.6-39.8)**	21.0	(17.3-24.7)
**Sodium (mg)**, mean (95%CI)	3406.9	(3340.6-3473.2)	3239.0	(3163.0-3314.9)	**3602.8**	**(3503.0-3702.6)**

95%CI: 95% confidence interval; variables adjusted for sex, age, income, and usual intake. Values in **bold** were significant (p<0.05).

The top 20 food sources contributing to energy intake from snacks of all study participants and separated by age groups are listed in Table 3. The five most common food sources for all participants contributed with 30.5% of the total energy intake from snacks: 11.8% cookies, 9.4% sugar-sweetened beverages, and 9.3% sweets and other desserts. Adolescents’ food sources accounted for 14.9% of cookies, 10.9% of sweets and other desserts, and 9.5% of breads. Young adults’ most common food sources were sugar-sweetened beverages (10.5%), croquettes (9.0%), and cookies (8.4%).

**Table 3:** Sources of energy intake from snacks consumed by adolescents (n=418) and young adults (n=218) living in the city of São Paulo. 2015 ISA-Capital.

Rank	Food groups	PCT	Food groups	PCT	Food groups	PCT
	Total sample		Adolescents		Young adults	
1	Cookies	11.8	Cookies	15.0	Sugar-sweetened beverages	10.5
2	Sugar-sweetened beverages	9.4	Sweets and other desserts	10.9	Croquettes	9.1
3	Sweets and other desserts	9.3	Breads	9.5	Cookies	8.4
4	Breads	8.9	Sugar-sweetened Beverages	8.5	Bread	8.3
5	Croquettes	6.7	Savory snacks	6.0	Sweets and other desserts	7.7
6	Crackers	5.4	Fruits	4.6	Crackers	6.4
7	Savory snacks	4.5	Croquettes	4.5	Cakes	6.2
8	Savory bakery pastries	4.4	Butter and margarine	4.4	Alcoholic beverages	5.2
9	Fruits	4.3	Crackers	4.4	Savory bakery pastries	4.9
10	Cakes	4.3	Milk	4.2	Fruits	4.1
11	Milk	4.0	Savory bakery pastries	4.0	Milk	3.7
12	Butter and margarine	3.3	Non-processed meats	2.7	Non-processed meats	2.9
13	Alcoholic beverages	3.1	Cakes	2.4	Savory snacks	2.9
14	Non-processed meats	2.8	Cheese	1.7	Cheese	2.1
15	Cheese	1.9	Processed meats	1.7	Yogurt	2.0
16	Yogurt	1.7	Chocolate powder	1.5	Butter and margarine	2.0
17	Processed meats	1.6	Nuts and seeds	1.4	Processed meats	1.5
18	Chocolate powder	1.3	Yogurt	1.4	Sandwiches	1.5
19	Sandwiches	1.3	Other preparations	1.3	Sugar	1.3
20	Sugar	1.2	Rice	1.2	Pasta	1.2
Total		91.2		91.1		91.7

PCT: percentage of energy contribution in total energy intake.

An examination of the contribution of snacks to the overall sample per sex, lifestyle behaviors, and weight status revealed non-significant findings, but leading to a positive hypothesized direction for snack consumption, as shown in Table 4. Females (OR 0.93, 95%CI 0.36-1.49) who met the current recommendations for PA (OR 0.75, 95%CI 0.25-1.25) scored higher in the Brazilian Healthy Index-Revised (2^nd^ tercile of OR 0.67, 95%CI 0.10-1.25 and 3^rd^ tercile OR 0.88, 95%CI 0.24-1.5) and had a higher intake of added sugars (3^rd^ tercile; OR 1.18, 95%CI 0.46-1.90), showing a high intake of snacks. Overweight individuals were less likely to consume snacks (OR -0.54, 95%CI -1.00 to -0.08).

**Table 4:** Snacks consumption, sex and lifestyle behaviors among adolescents (n=418) and young adults (n=218). 2015 Health Survey of São Paulo with focus on Nutrition (2015 ISA-Nutrition).

	Overall sample		
	OR (SE)		95%CI
Sex (reference: male)			
Female	0.9	0.3	0.4-1.5
Weight status (reference non-overweight)			
Overweight/obese	-0.5	0.2	-1.0 to -0.1
PA (reference: does not meet recommendations for PA)			
Meets the recommendations for PA	0.8	0.3	0.3-1.3
BHEI-R (reference: 1^st^ tercile)			
2^nd^ tercile	0.7	0.3	0.1-1.3
3^rd^ tercile	0.9	0.3	0.2-1.5
Added sugars (reference: 1^st^ tercile)			
2^nd^ tercile	0.4	0.3	-0.2-1.1
3^rd^ tercile	1.2	0.4	0.5-1.9

BHEI-R: Brazilian Healthy Eating Index-Revised; 95%CI: 95% confidence interval; OR: *Odds Ratio*; PA: physical activity; SE: standard error. Adjusted for age groups, educational level, per capita income, presence of chronic non-communicable diseases, race, screen time, sleeping time, and total energy and sodium intake. Significant values are bolded (p<0.05).

## DISCUSSION

This study tried to understand the snacking behaviors of adolescents and young adults, as well as their daily energy, nutrients and food intakes. The findings indicated that snacking behaviors differed between adolescents and young adults. Thus, they offer valuable data to support health policies and behavioral-changing strategies to promote healthy weight status and lifestyle behaviors. Snacking can provide new opportunities to meet daily nutritional needs and numerous factors influence the intake by adolescents and young adults.^9^ Similar to our results, the Canadian Community Health Survey (CCHS) found a high prevalence of snacking among adolescents and young adults, i.e., more than 50%.^25^ In contrast to the Canadian study, young adults have a slightly higher frequency of snack intake compared to adolescents. Young adults consumed more energy from snacks than adolescents, especially in mid-morning and night snacks. Alternatively, adolescents had more energy intake in afternoon-snacks. Moreover, adolescents and young adults obtained about one-quarter of their daily intake from breads, sugar-sweetened beverages, and sweets in snack times, corroborating another nationwide study conducted with 25,753 individuals older than 10 years,^26^ suggesting that these age groups are consuming these foods away from home. These results are important from the public health perspective. Snacks are related to the total energy intake and may differ according to some aspects, including the location where they are consumed. Therefore, this must be taken into account when thinking of health policies and behavior-change strategies. For example, evidence has shown that better cooking skills are associated with support in meal preparation and healthy eating, with great impact on diet quality.

Results suggest 36 to 256kcal/day intake in snacks. This variation of energy intake in snacks was associated with the time of the day when they were consumed: morning. afternoon, or night snack. This may suggest that energy intake from snack consumption can help reduce total daily energy intake. Evidence supports that other meals (i.e., breakfast, lunch, and dinner) were not associated with high-energy intake. This analysis allowed adjustment for potential confounders, such as total energy intake, as well as were looked for consumption time of snacks, being consistent with evidence showing that the energy yielded by snacks is not compensated for in the subsequent meal and leas consistently to higher total daily intake.^27^ Reports of mixed results on the effects of snacks on energy intake, can be attributed to broad variations in the definitions used for snacks, allowing this inconsistency.^11^ ^,^ ^28^ Concurrently, portion size served at one meal or in-between meals is positively associated with total energy intake.^27^ Hence, a low-energy intake in morning snacks and a higher-energy in afternoon snacks may influence energy intake at lunch and dinner, respectively, with improvement on overall diet quality. While further evidence is needed, consuming energy from snacks may help balance energy intake from other meals.

Evidence has shown that low-income families face some challenges on making healthy food choices, such as limited access to fruit and vegetables, whole-grains, and reduced-fat milk and dairy food sources within their food environment (at home and away from home). These challenges are not only limited to understanding what is (un)healthy food choices. For example, a qualitative study conducted with 83 adolescents of low-income status provided a simplistic view on healthy eating, i.e., dichotomizing foods into bad and good, with a prevalence of overweight and obesity of 28.9%, with 42.1% of adolescents not considering their diet healthy, given that they report consuming food sources with sodium, saturated fats, and added sugars.^29^ The reasons for choosing these foods are not only related to their preferences, but also to access and availability, distance to food stores, cooking skills, and time available to prepare their meals.^30^ The complex and numerous challenges faced by low-income individuals required the development and implementation of innovative public health and behavior-changing strategies; the findings of the current study reinforce that further studies should try and understand the need for cultural tailoring of interventions found to be effective in making smart snack choices.

 One of the strength points of this study is the fact that it was population-based and conducted in a large urban center. It is well known that representative surveillance data are important to identify broader dietary patterns, but limitations should be taken into account. This analysis is based on a 24hRs and relies on self-reported data. As a cross-sectional study, a casual inference is not possible.

In conclusion, health policies and behavior-changing strategies aiming to improve the quality of snacks might broaden the aspects beyond energy-nutrient knowledge, reaching a more culture-tailored intervention. For example, focusing on the ability of choosing low-cost healthy foods and beverages and on learning to prepare healthy meals.
